# Comparative phylogeography between two generalist flea species reveal a complex interaction between parasite life history and host vicariance: parasite-host association matters

**DOI:** 10.1186/s12862-015-0389-y

**Published:** 2015-06-10

**Authors:** Luther van der Mescht, Sonja Matthee, Conrad A. Matthee

**Affiliations:** Department of Conservation Ecology and Entomology, Stellenbosch University, Private Bag ×1, Matieland, 7602 Stellenbosch, South Africa; Evolutionary Genomics Group, Department of Botany and Zoology, Stellenbosch University, Private Bag ×1, Matieland, 7602 Stellenbosch, South Africa

**Keywords:** Host specificity, Ectoparasite, Life history, Phylogeography, Siphonaptera, Vicariance

## Abstract

**Background:**

In parasitic taxa, life history traits such as microhabitat preference and host specificity can result in differential evolutionary responses to similar abiotic events. The present study investigates the influence of vicariance and host association on the genetic structure of two generalist flea species, *Listropsylla agrippinae,* and *Chiastopsylla rossi*. The taxa differ in the time spent on the host (predominantly fur vs. nest) and level of host specificity.

**Results:**

A total of 1056 small mammals were brushed to collect 315 fleas originating from 20 geographically distinct localities in South Africa. Phylogeographic genetic structure of *L. agrippinae* and *C. rossi* were determined by making use of 315 mitochondrial *COII* and 174 nuclear *EF1-α* sequences. Both parasites show significant genetic differentiation among the majority of the sampling sites confirming limited dispersal ability for fleas. The generalist fur flea with a narrower host range, *L. agrippinae,* displayed geographic mtDNA spatial genetic structure at the regional scale and this pattern is congruent with host vicariance. The dating of the divergence between the *L. agrippinae* geographic clades co-insides with paleoclimatic changes in the region approximately 5.27 Ma and this provides some evidence for a co-evolutionary scenario. In contrast, the more host opportunistic nest flea, *C. rossi,* showed a higher level of mtDNA and nDNA spatial genetic structure at the inter-populational scale, most likely attributed to comparatively higher restrictions to dispersal.

**Conclusions:**

In the present study, the evolutionary history of the flea species could best be explained by the association between parasite and host (time spent on the host). The phylogeographic pattern of the fur flea with a narrower host range correspond to host spatial genetic structures, while the pattern in the host opportunistic nest flea correspond to higher genetic divergences between sampling localities that may also be associated with higher effective population sizes. These findings suggest that genetic exchange among localities are most likely explained by differences in the dispersal abilities and life histories of the flea species.

**Electronic supplementary material:**

The online version of this article (doi:10.1186/s12862-015-0389-y) contains supplementary material, which is available to authorized users.

## Background

Paleoclimatic events have been put forward as one of the main mechanisms causing speciations and extinctions of evolutionary lineages [1–4]. The differences in ecology and life history of taxa, however, often result in differential species-specific responses to similar climatic events [5–7] and a simple explanation describing speciation processes hardly exists. For parasitic taxa the situation is further perplexed by complex life cycles, host biogeography, and the level of parasite-host associations [8–16]. Although some progress has been made regarding this topic [17–20] more quantifiable data are needed to make more accurate predictions on for example the factors affecting range expansions and connectivity among populations. For many parasitic taxa, this is desperately needed from a disease ecology perspective [21, 22].

**Fig. 1:**
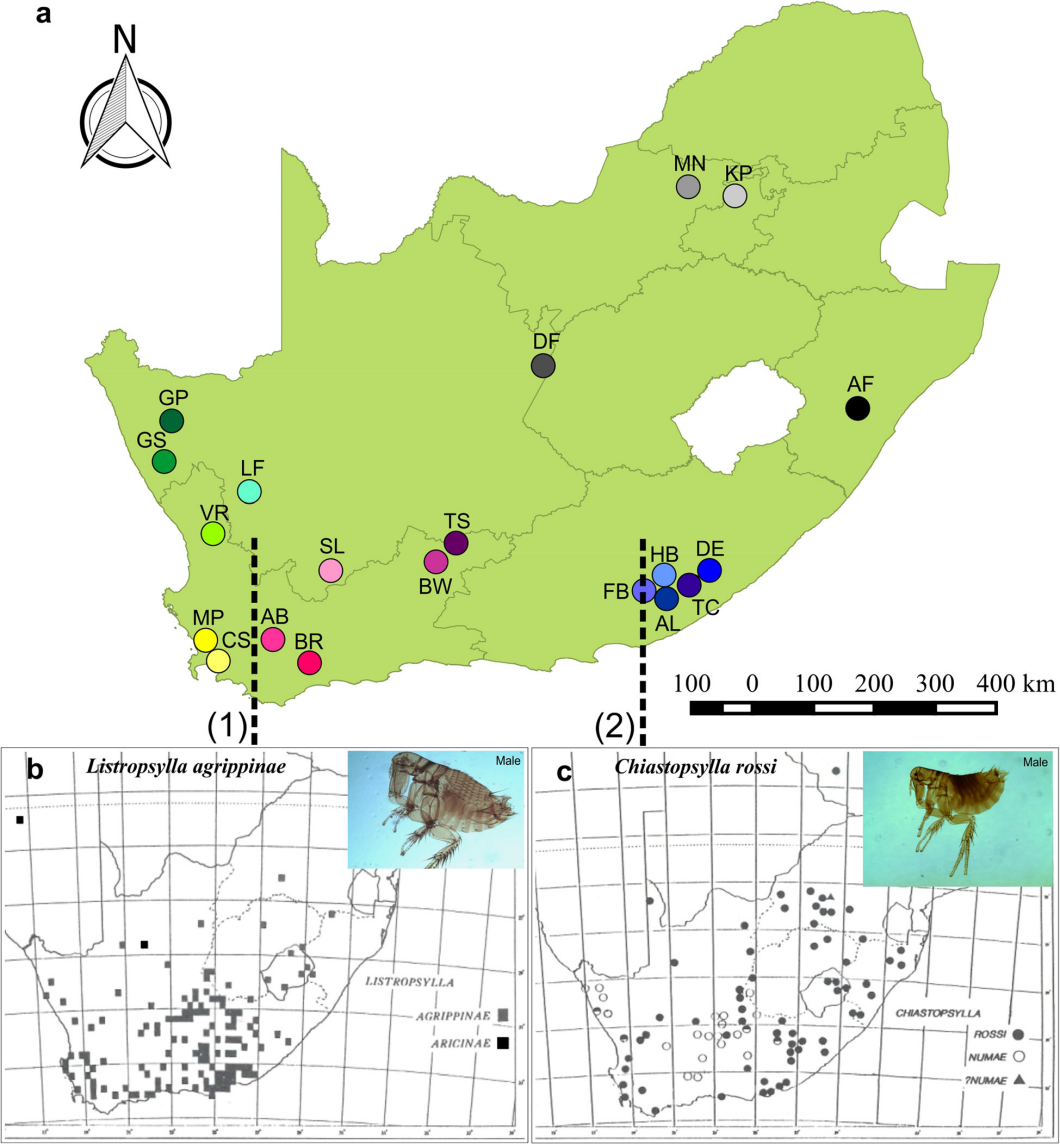
Map of sampling localities. Map indicating **a** localities sampled with their respective codes indicated as in Table 5. The vicariant breaks in South Africa (1) winter and aseasonal rainfall break and (2) xeric and mesic biomes are indicated by white dashed lines, **b** the mapped distribution of *L. agrippinae* and **c** *C. rossi* are taken directly from [34]

In southern Africa, several phylogeographic studies have been performed on small vertebrate taxa (for example see [23–29]). From these studies, paleoclimatic changes are once again suggested as one of the main drivers of evolution. Congruent phylogeographic patterns among lizards, rodents, shrews and elephant shrews also suggested the existence of vicariant biogeographic barriers in the region (for review see [7, 30]). Of particular relevance to the present study is the support for regional vicariance found in *Rhabdomys* [29]*, Myosorex* [27], *Otomys* [28] and *Micaelamys* [26]. These small mammals show distinct genetic clades that can be associated with the xeric western succulent biomes and the more mesic eastern predominantly grassland biome of southern Africa (Fig. 1a; Additional file 1; also see [7]). In addition, *Rhabdomys, Myosorex* and *Micaelamys* show differentiation to a larger or lesser extent between the winter rainfall zone and the aseasonal rainfall zone in the Cape Floristic Region (Fig. 1a; Additional file 1; also see [30]). From these studies it is evident that the diversification of small mammals in the region is driven by complex interactions between life history, climate and topographic barriers.

The effect of vicariant barriers on the evolution of parasites occurring in southern Africa is virtually unknown. An exception to this is provided by [13], who recently indicated partial congruence in spatial genetic structure between *Rhabdomys* and its species specific lice, *Polyplax*. Distinct genetic clades in the parasites and in the hosts supported the documented mesic eastern and xeric western divide in the subregion (see above). Co-evolution analyses, however, failed to support a strong signal of geographic co-differentiation between parasite and host and the authors suggested that the resultant pattern is due to the synergistic effects of parasite traits (host specificity), host-related factors (the vagility and social behavior of *Rhabdomys*) and the biogeography (vicariance) of the region [13]. To expand on these findings, we here present phylogeographic data on two generalist flea species occurring on *Rhabdomys* and other small mammal species in southern Africa.

Individual flea species are normally adapted to utilize a variety of hosts in the environment and are all obligatory blood feeders of endothermic vertebrates [31, 32]. The life cycle consists of four stages (egg, larva, pupa, and adult) and fleas are dependent on the nest environments of the hosts to variable extent [31]. Immatures develop entirely in the off-host environment while adult stages have to spend some time on the host to obtain a blood meal. The length of time that is spent on the host, however, varies between flea taxa [31–33]. This observation has provided an opportunity to coarsely classify fleas according to their microhabitat preference as either a “fur” (adult stage spend more time on the host) or a “nest” flea (adults spend more time in the nest/burrow of the host; [31–33]). These differences can have profound effects on the ability of the flea species to disperse over the landscape and we therefore predict that fur fleas will show more phylogeographic congruence with the host/s (adult fleas spend more time on the host and can thus disperse over the landscape) and nest fleas will show more restricted gene flow across the landscape (since all juveniles and the adults spend the majority of their life cycle off the host and have limited dispersal capabilities).

To test the hypothesis that fleas with different levels of host association will differ in spatial genetic structure, the fur flea, *Listropsylla agrippinae,* and the nest flea, *Chiastopsylla rossi* [34, 35] were selected. Both *L. agrippinae* and *C. rossi* occur widespread throughout southern Africa (Fig. 1) [35, 36] and span at least two well documented vicariant biogeographic barriers in the region (Fig. 1; [7, 30]). Apart from differences in the duration of time adults spend on the host, the two species also differ in the level of host specificity (niche breath). *Listropsylla agrippinae* has two principle host taxa (*Rhabdomys* spp. and *Myotomys unisulcatus*) whereas *C. rossi* has at least four main host taxa (*Rhabdomys* spp., *Otomys irroratus*, *M. unisulcatus* and *Tatera brantsii*) documented [34, 35, 37]. It has been suggested that host specificity may influence the level of intraspecific genetic divergences since more generalist parasite species will show a higher level of intraspecific genetic variation enabling them to infest a broader host range [38–40]. Furthermore, microhabitat preference (specifically referring to fur vs nest) is not always related to host specificity, meaning that fleas may differ in host specificity irrespective of microhabitat preference (see [32, 34, 36]). If niche breath and time spent on the host affect 1) genetic diversity of the parasite and 2) congruence in phylogeographic structure between parasites and hosts [38–40], we predict that *L. agrippinae,* when compared to *C. rossi,* will show lower levels of intraspecific genetic diversity and also more similarities to the vicariant patterns of the hosts they infest. Genetic distances among individuals from different sampling sites are expected to be higher for the nest flea, *C. rossi,* when compared to the fur flea, *L. agrippinae,* since the latter can utilize the host for dispersion. By considering the interplay between life history and geography, the present study should add valuable information needed to explain some of the evolutionary processes that shape ectoparasite distribution and diversity.

## Results

### Parasite prevalence and distribution

**Fig. 2:**
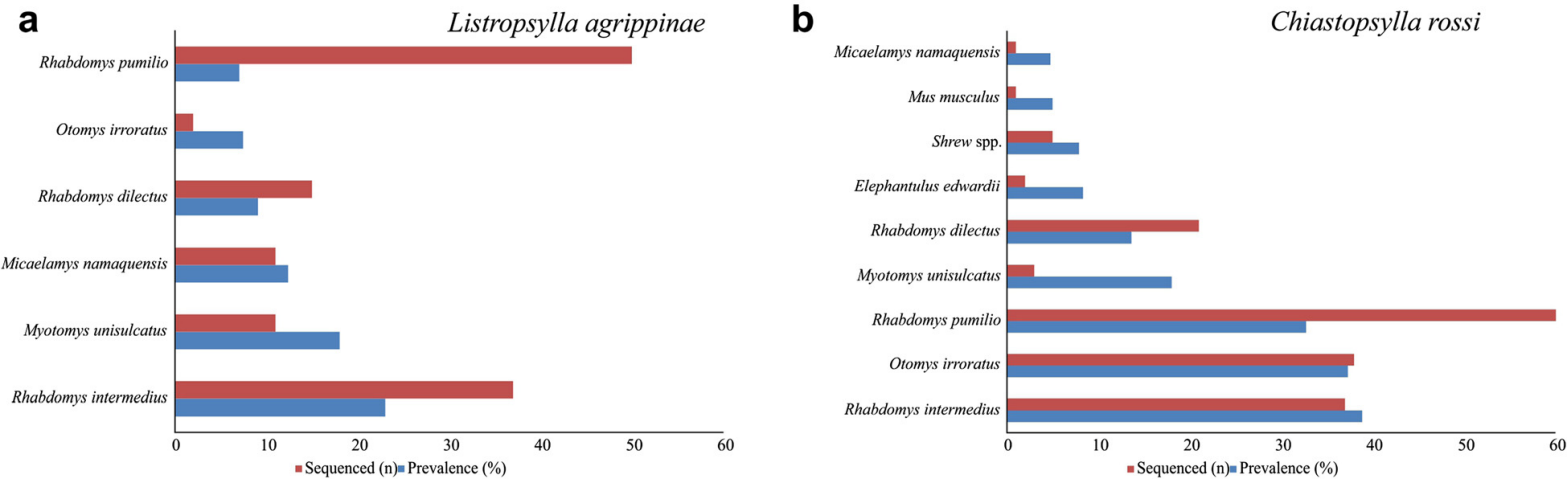
Barplots of number of individuals sequenced and prevalence. The number of individuals sequenced and prevalence of **a** *L. agrippinae* and **b** *C. rossi* sampled from different small mammal host species at 20 localities throughout South Africa

Both *R. pumilio* and *R. dilectus* were trapped at a contact zone, Fort Beaufort (also see [29]), and represent the only locality where more than one *Rhabdomys* species was trapped (see Additional file 2). *Listropsylla agrippinae* were recorded at lower prevalence at the majority of localities when compared to *C. rossi* (Fig. 2; Additional file 2). *Listropsylla agrippinae* was found on six host species and was most prevalent on *R. intermedius* (23.02 %) (Fig. 2a). *Chiastopsylla rossi* was found on nine host species, but most prevalent on *R. intermedius* (38.89 %) and *O. irroratus* (37.31 %) compared to the other 4 host taxa (Fig. 2b). Individuals selected for sequencing were representative of the various host species trapped at each locality (Fig. 2; Additional file 2).

### Characteristics of molecular markers

Mitochondrial *COII* data for 126 *L. agrippinae* and 189 *C. rossi* specimens were generated and attempts were made to generate a similar nuclear *EF1-α* data set. Nuclear amplification was less successful but we nonetheless generated 94 *L. agrippinae* and 254 *C. rossi* nuclear alleles (Table 1) [GenBank: KR 263182–263844]. Haplotype and nucleotide diversity values were lower for *L. agrippinae* when compared to *C. rossi* for mitochondrial *COII* and nuclear *EF1-α* data (Table 1). Unexpectedly, the nuclear *EF1-α* data for both species resulted in higher diversity estimates in terms of haplotypic and nucleotide diversity when compared to the mitochondrial *COII* data (Table 1).

**Table 1:** Summary statisitics

	Sequences/Alleles	Total haplotypes	Singleton haplotypes	bp	*P*	π ± SD	*h* ± SD
COII							
*L. agrippinae*	126	35	13	576	62	0.013 ± 0.003	0.925 ± 0.015
*C. rossi*	189	51	26	513	69	0.021 ± 0.006	0.958 ± 0.005
EF1-α							
*L. agrippinae*	47/94	54	42	593	88	0.010 ± 0.003	0.946 ± 0.017
*C. rossi*	127/254	177	90	579	184	0.022 ± 0.004	0.994 ± 0.001

Summary statistics for the mitochondrial DNA (*COII*) and nuclear intron (*EF1-*α) markers sequenced from *L. agrippinae* and *C. rossi.* The number of sequences/alleles, total number of haplotypes, total number of singleton haplotypes, fragment length (bp), number of polymorphic sites (*P*), nucleotide diversity (π) and haplotype diversity (*h*) is indicated

### Phylogeographic analyses

#### Listropsylla agrippinae

**Fig. 3:**
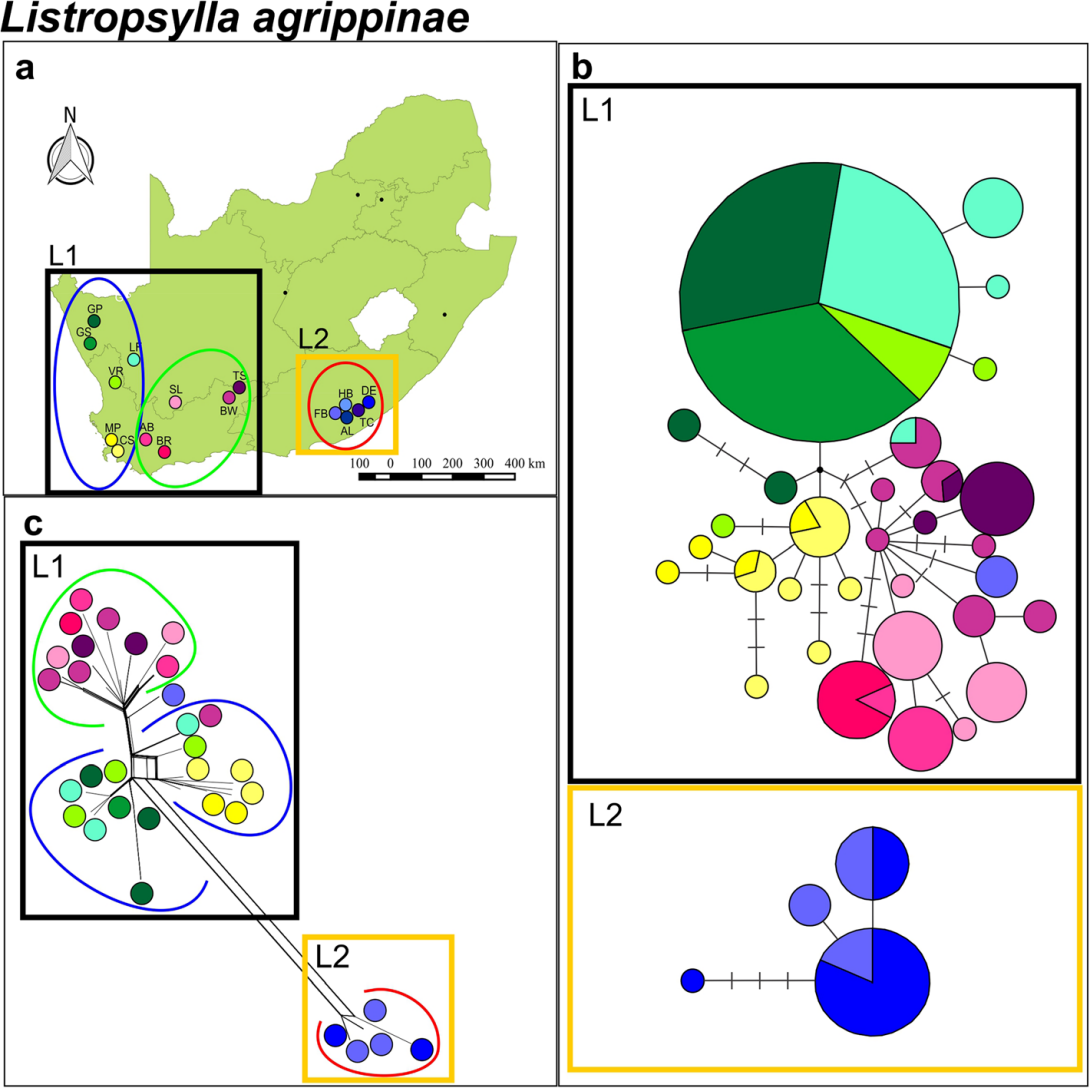
*Listropsylla agrippinae* mitochondrial *COII.* **a** A map of geographical distribution of sampling localities in South Africa for *L. agrippinae*. Bayesian analysis of population structure (BAPS) subclusters are indicated by green, blue and red circles. **b** Statistical parsimony mitochondrial *COII* haplotype network colour coded according to sampling locality. Circle size depicts frequency, branches depict single mutational steps, small black circles display intersections and cross hatching indicate missing haplotypes/mutational steps. Clades are bound by black (L1) and deep yellow (L2) line boxes. **c** Neighbour-Net phylogenetic network for *L. agrippinae* labelled according to haplotype groupings from statistical parsimony results and are bound by black (L1) and deep yellow (L2) line boxes. Bayesian analysis of population structure (BAPS) subclusters are indicated by green, blue and red lines

**Fig. 4:**
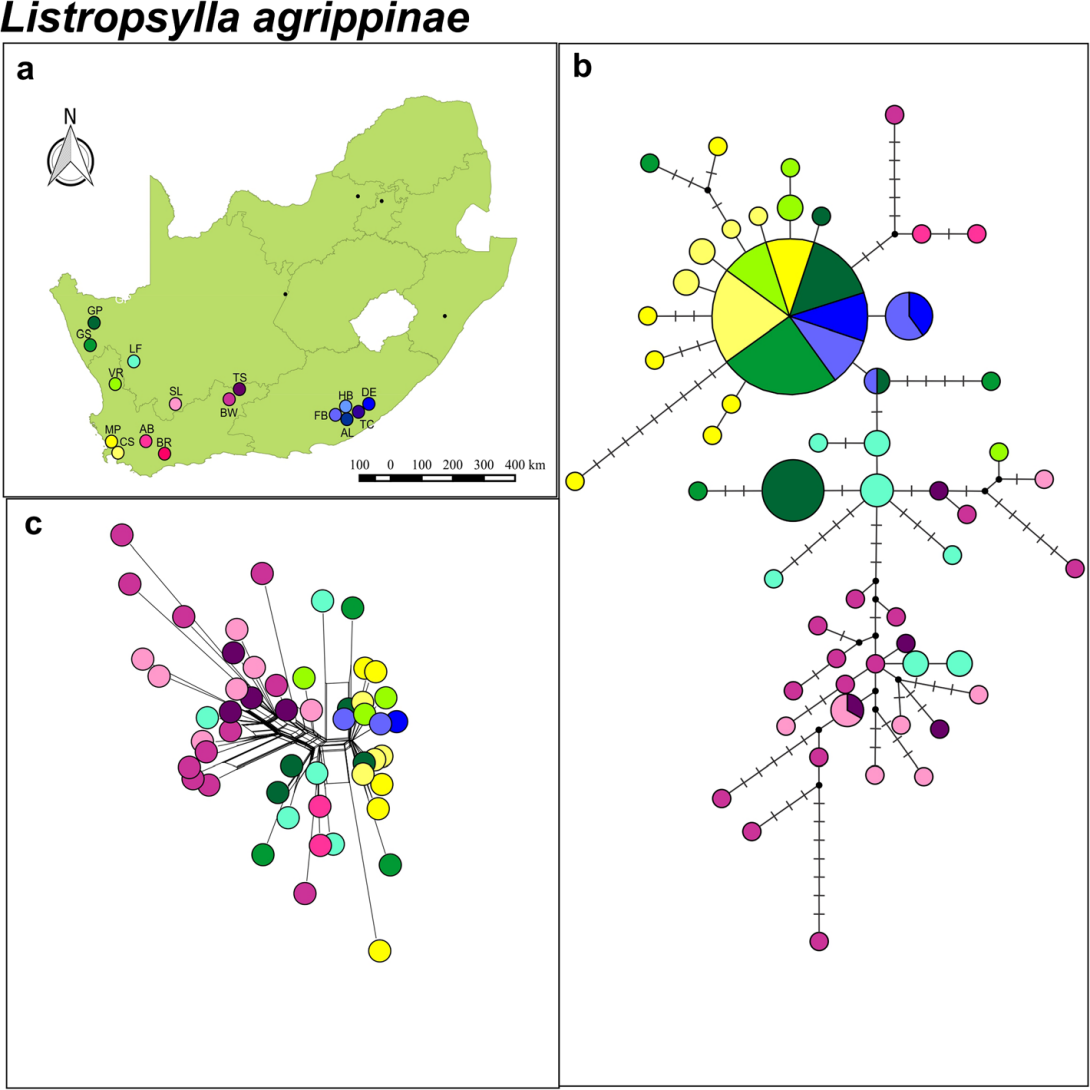
*Listropsylla agrippinae* nuclear *EF1-α.* **a** A map of geographical distribution of sampling localities in South Africa for *L. agrippinae*. **b** Statistical parsimony nuclear *EF1-α* haplotype network colour coded according to sampling locality. Circle size depicts frequency, branches depict single mutational steps, small black circles display intersections and cross hatching indicate missing haplotypes/mutational steps. **c** Neighbour-Net phylogenetic network for *L. agrippinae*

TCS statistical parsimony analysis based on the mitochondrial *COII* data showed two distinct clusters (L1 and L2; Fig. 3a and b) that could not be connected within the 95 % confidence interval. These two clusters are separated by an average corrected mtDNA sequence divergence of 3.02 % (±0.36) and this large differentiation is further supported by the Neighbor-Net tree (Fig. 3c). The clusters correspond to the xeric western (L1) and eastern mesic (L2) zones of the country. The BAPS analysis corroborated the two clusters detected by TCS and the Neighbor-Net tree, and also indicate some additional substructure within cluster L1 (*P* = 1.00; log likelihood of optimal partition = − 1179.80) (Fig. 3a and c). These two subclusters loosely correspond to the winter and aseasonal rainfall divide (Fig. 1). The spatial genetic patterns were less pronounced when the nuclear *EF1-α* data is compared to the mitochondrial DNA network (Figs. 3 and 4). There is some evidence for regional clustering as indicated by the grouping of similarly shaded colors in the parsimony network and Neighbor-Net tree (Fig. 4) but more importantly, however, the most common DNA allele is shared between the far eastern and far western side of the sampling distribution. Mantel tests for mitochondrial *COII* data indicated that there were weak isolation by distance when calculated for all localities (*r* = 0.11; *P* = 0.00) and when calculated for each cluster L1 (*r* = 0.11; *P* = 0.01) and L2 (*r* = 0.16; *P* = 0.01) separately. The time of divergence between the two main clusters within *L. agrippinae* date back to the late Miocene which was estimated to be 5.27 Ma (95 % HPD interval: 2.31, 9.58 Ma).

**Fig. 5:**
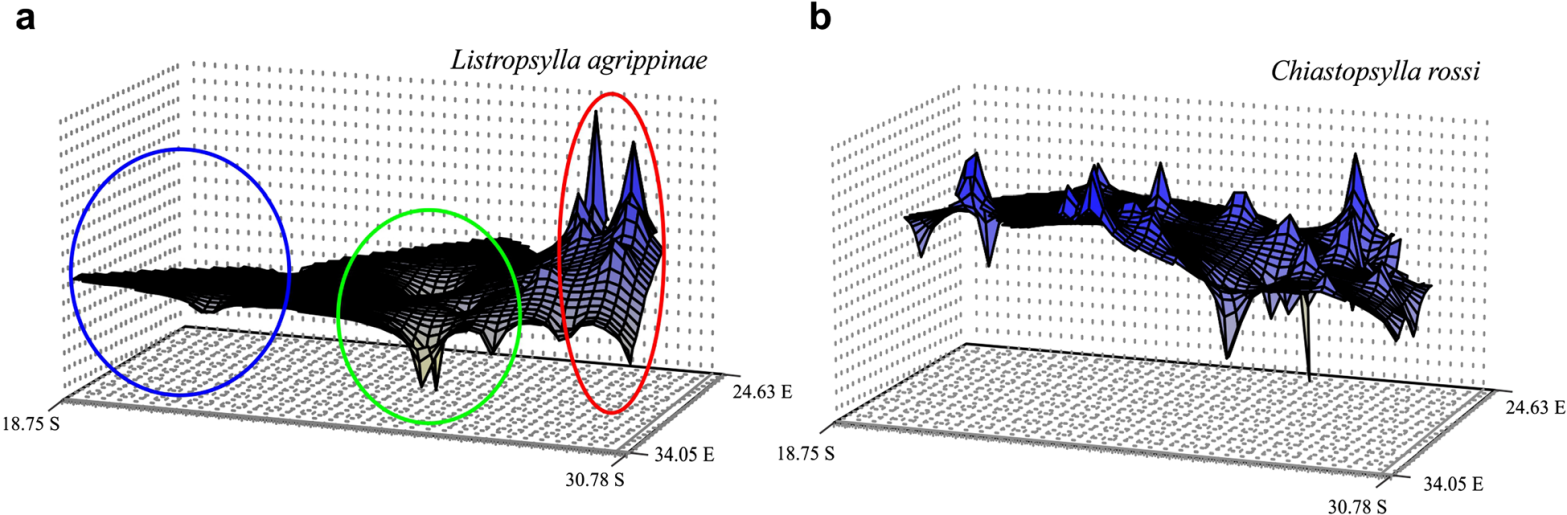
Genetic landscape interpolation plots. Genetic landscape interpolation plot for total distribution of **a** *L. agrippinae*, and **b** *C. rossi*

Genetic landscape interpolation surface plots for the *COII* data indicated that there were marked differences between some sampling localities when the distribution of genetic diversity found at each is compared across the landscape (Fig. 5a). The graphical representation suggests that the area of greatest genetic diversity was found in the contact zone close to the ‘Bedford gap’ (Fig. 5a). Fixation index values of mitochondrial *COII* for *L. agrippinae* were significant at all levels and the highest level of differentiation was recovered among subclusters (60.00 % of variation) (Table 2). Fixation index values of nuclear *EF1-α* for *L. agrippinae* were significant at all levels but in this instance the highest level of variation was recovered within localities (63.26 % of variation) (Table 2). Pairwise *Φst* values among sampling localities showed that the majority of sampling sites are significantly differentiated from each other for both the nuclear and mtDNA data (Table 3).

**Table 2:** Hierarchical analysis of molecular variance

	Number of groups	Fixation indices			Percentage variation		
		*F*_CT_	*F*_SC_	*F*_ST_	Among subclusters	Among localities within subclusters	Within localities
*L. agrippinae*							
COII	3	**0.600**	**0.439**	**0.775**	60.00	17.54	22.45
EF1-α	3	**0.239**	**0.169**	**0.367**	23.91	12.83	63.26
*C. rossi*							
COII	6	**0.513**	**0.382**	**0.699**	51.29	18.63	30.08
EF1-α	6	**0.145**	**0.429**	**0.512**	14.51	36.70	48.79

Hierarchical analysis of molecular variance (AMOVA) of mitochondrial DNA (*COII*) and nuclear intron (*EF1-*α) in two flea species for BAPS subclusters. Fixation index values are given for the hierarchical levels examined: *F*_*ST*_ within localities; *F*_*CT*_ among subclusters; and *F*_*SC*_ among localities within subclusters. Significant (p < 0.05) tests are indicated in bold

**Table 3:** *Listropsylla agrippinae* pairwise *Φst* values

Localities	GP	GS	LF	VR	MP	CS	SL	AB	BR	TS	BW	FB	DE
GP		**0.18**	**0.26**	**0.35**	**0.26**	**0.41**	**0.46**	**0.62**	-	**0.55**	**0.31**	**0.43**	**0.38**
GS	**0.18**		**0.27**	0.04	0.00	**0.04**	**0.37**	**0.42**	-	**0.43**	**0.26**	0.05	−0.02
LF	**0.17**	0.09		**0.35**	**0.31**	**0.44**	**0.23**	**0.41**	-	0.21	**0.12**	**0.41**	**0.37**
VR	0.12	**0.23**	0.13		0.03	**0.16**	**0.40**	0.63	-	**0.54**	**0.26**	**0.24**	0.15
MP	**0.63**	**0.88**	**0.72**	**0.50**		**0.04**	**0.37**	**0.40**	-	**0.42**	**0.28**	0.04	−0.03
CS	**0.59**	**0.77**	**0.67**	**0.49**	0.07		**0.52**	**0.78**	-	**0.69**	**0.36**	**0.25**	0.15
SL	**0.65**	**0.75**	**0.70**	**0.60**	**0.71**	**0.71**		**0.30**	-	−0.06	0.03	**0.46**	**0.42**
AB	**0.71**	**0.88**	**0.77**	**0.64**	**0.75**	**0.76**	**0.27**		-	0.55	0.17	**0.84**	0.81
BR	**0.85**	**1.00**	**0.89**	**0.86**	**0.93**	**0.88**	**0.71**	**0.77**		-	-	-	-
TS	**0.77**	**0.92**	**0.82**	**0.77**	**0.85**	**0.82**	**0.51**	**0.66**	**0.90**		−0.06	**0.68**	**0.64**
BW	**0.52**	**0.60**	**0.56**	**0.42**	**0.57**	**0.59**	**0.13**	**0.30**	**0.59**	**0.39**		**0.30**	**0.26**
FB	**0.59**	**0.61**	**0.62**	**0.46**	**0.52**	**0.60**	**0.59**	**0.50**	**0.66**	**0.64**	**0.51**		−0.26
DE	**0.91**	**0.97**	**0.93**	**0.91**	**0.93**	**0.92**	**0.90**	**0.92**	**0.97**	**0.95**	**0.85**	**0.25**	

Pairwise *Φst* values among *L. agrippinae* sampled localities for mitochondrial DNA (*COII*) below and nuclear intron (*EF1-α*) above the diagonal. Significant values (p < 0.05) are highlighted in bold. (Locality codes as in Table 5)

#### Chiastopsylla rossi

**Fig. 6:**
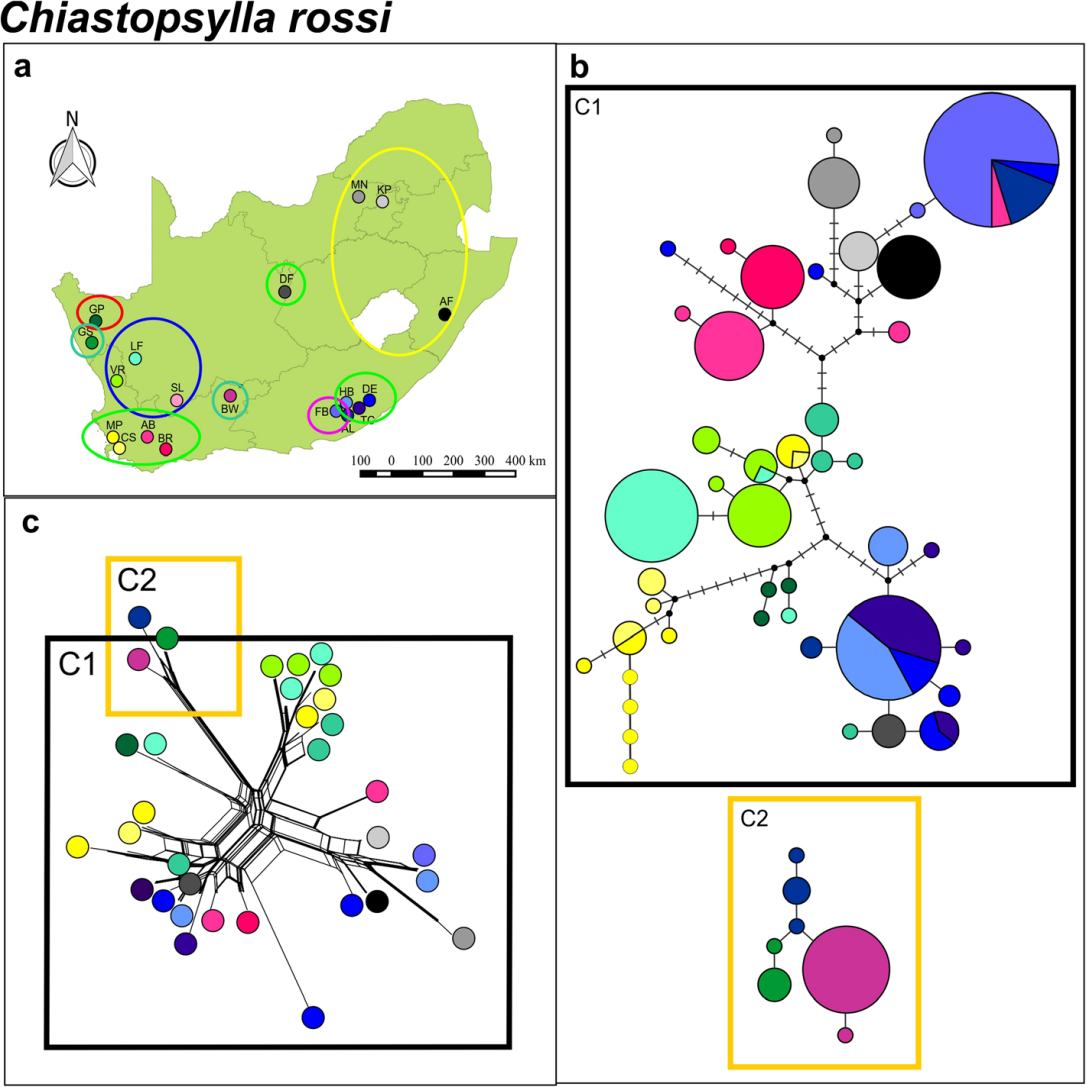
*Chiastopsylla rossi* mitochondrial *COII.* **a** A map of geographical distribution of sampling localities in South Africa for *C. rossi*. Bayesian analysis of population structure (BAPS) revealed six subclusters indicated by different colours. **b** Statistical parsimony mitochondrial *COII* haplotype network colour coded according to sampling locality. Circle size depicts frequency, branches depict single mutational steps, small black circles display intersections and cross hatching indicate missing haplotypes/mutational steps. Clusters are bound by black (C1) and deep yellow (C2) line boxes. **c** Neighbour-Net phylogenetic network for *C. rossi* labelled according to haplotype groupings from statistical parsimony results and are bound by black (C1) and deep yellow (C2) line boxes

**Fig. 7:**
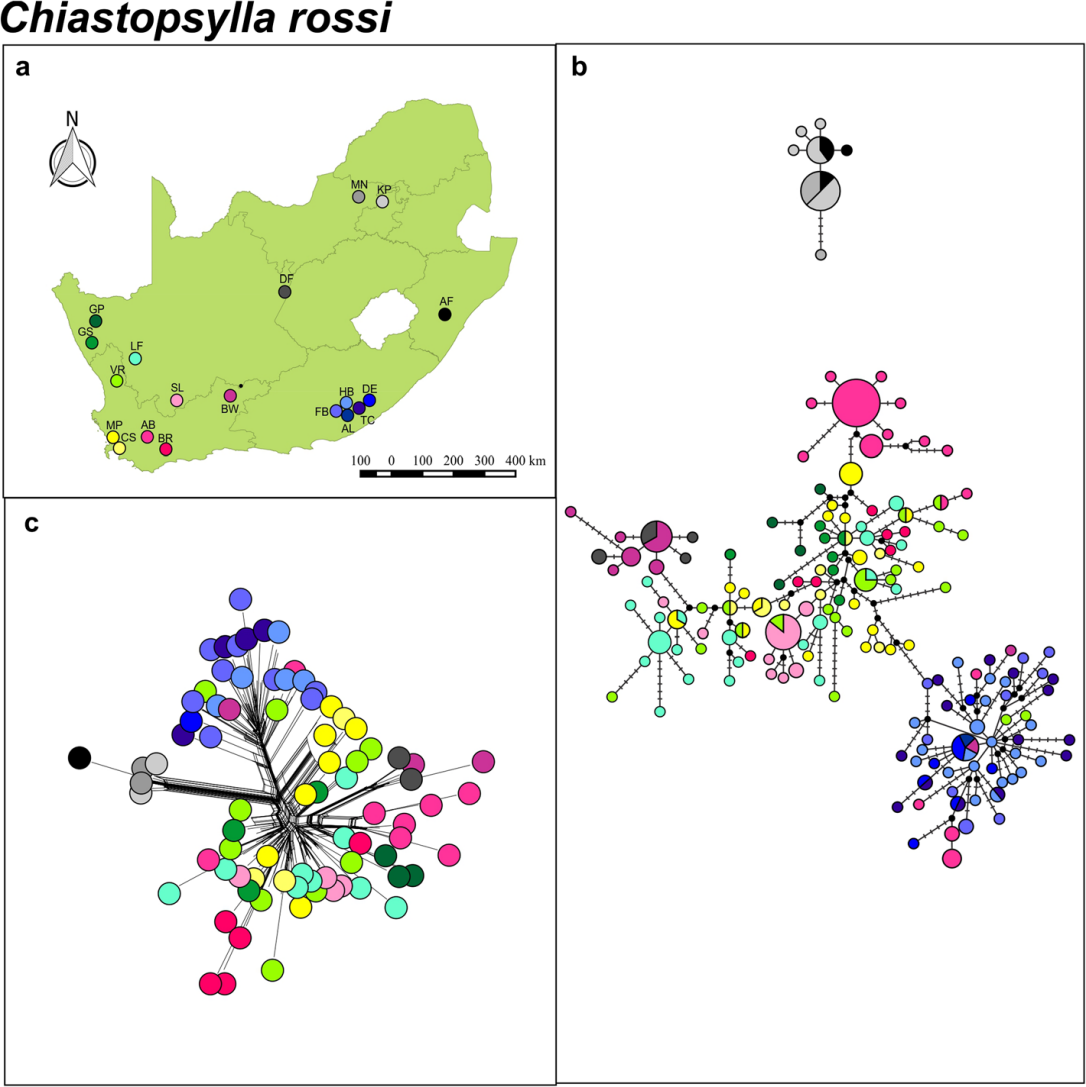
*Chiastopsylla rossi* nuclear *EF1-α.* **a** A map of geographical distribution of sampling localities in South Africa for *C. rossi*. **b** Statistical parsimony nuclear *EF1-α* haplotype network colour coded according to sampling locality. Circle size depicts frequency, branches depict single mutational steps, small black circles display intersections and cross hatching indicate missing haplotypes/mutational steps. **c** Neighbour-Net phylogenetic network for *C. rossi*

TCS analysis based on mitochondrial *COII* data showed a remarkably different genetic pattern for *C. rossi* when compared to *L. agrippinae.* The majority of the localities sampled are characterized by unique divergent haplotypes (Fig. 6a and b), and although two distinct genetic clusters separated by an average sequence divergence of 3.01 % (±0.32) were also obtained for this species (C1 and C2; Fig. 6b), it was not congruent with vicariant host patterns (Fig. 6). Instead, limited haplotype sharing was evident among distant sampling sites and the majority of populations were characterized by unique/closely related locality specific haplotypes (Fig. 6a and b). The lack of clear geographic structure is further supported by the Neighbor-Net tree (Fig. 6c) and moreover by the BAPS analyses suggesting at least six distinct subclusters present within *C. rossi* (*P* = 0.99; log likelihood of optimal partition =−1862.85). The spatial genetic pattern of the nuclear *EF1-α* network and the Neighbor-Net tree suggest more lineage sharing across the landscape when compared to the mitochondrial DNA *COII* data (Figs. 6 and 7). Similarly to *L. agrippinae,* the nuclear data also indicate some level of population differentiation (closely related haplotypes confined to single localities). The nuclear TCS analyses suggest a distinct clade comprising individuals from KP, MN and AF and this assemblage is supported as one of the six mtDNA BAPS subclusters. This subcluster is mainly found in the north eastern part of South Africa, and although it may have some biological meaning, the Neighbor-Net analysis (Fig. 7c) show that comparatively the level of differentiation is low. The majority of the subclusters identified by the mtDNA and nDNA data do not corresponded to any of the known biogeographic breaks previously documented for the majority of the host species and in fact are scattered across the landscape (Fig. 6a). Mantel tests indicated that there were virtually no isolation by distance within *C. rossi* when calculated for all localities (*r* = 0.06; *P* = 0.00).

The scattered distribution coupled to high inter-populational genetic variation in *C. rossi* is supported when the genetic landscape surface plot is considered. In contrast to *L. agrippinae*, *C. rossi* show several genetic discontinuities (peaks) throughout the total sampling distribution (Fig. 5b). Fixation index values of the mitochondrial *COII* for *C. rossi* were significant at all levels and the highest level of differentiation was recovered among subclusters (51.29 % of variation; Table 2). The highest level of differentiation for the *EF1-α* was recovered within localities (48.79 % of variation; Table 2). Pairwise *Φst* values for the mitochondrial and nuclear data also showed significant differentiation among the majority of sampling localities and the highest level of differentiation was again generally detected between KP, MN and AF and the remainder of the populations (Table 3).

## Discussion

Parasites are generally considered to have a high mutation rate, small effective population size and a limited dispersal ability. From this, it is predicted that pronounced spatial genetic structuring will be evident among sampling sites due to reduced gene flow and increased genetic drift [8, 41–43]. Overall, the results presented for the two generalist parasite species in this study conform to these suggestions. Most of the geographically distinct populations sampled show significant differentiation among sampling sites at both the mtDNA and nDNA level (Tables 3 and 4) a scenario attributed to reduced gene flow across the landscape (also see [38, 44]).

**Table 4:** *Chiastopsylla rossi* pairwise *Φst* values

Localities	GP	GS	LF	VR	MP	CS	SL	AB	BR	BW	HB	AL	FB	TC	DE	AF	DF	MN	KP
GP		**0.47**	**0.43**	**0.56**	**0.62**	**0.64**	**0.34**	**0.47**	**0.59**	**0.50**	**0.39**	**0.45**	**0.50**	**0.59**	**0.52**	**0.82**	**0.43**	**0.73**	**0.44**
GS	**0.31**		**0.08**	**0.42**	**0.60**	**0.65**	**0.04**	**0.43**	**0.65**	**0.60**	0.06	**0.22**	**0.49**	**0.73**	**0.52**	**0.84**	0.04	**0.81**	**0.43**
LF	**0.50**	**0.88**		**0.20**	**0.57**	**0.58**	**0.05**	**0.37**	**0.56**	**0.49**	**0.10**	**0.21**	**0.51**	**0.56**	**0.53**	**0.70**	0.03	**0.67**	**0.46**
VR	**0.50**	**0.89**	**0.46**		**0.64**	**0.71**	**0.15**	**0.40**	**0.71**	**0.65**	**0.27**	**0.44**	**0.56**	**0.74**	**0.60**	**0.82**	**0.26**	**0.80**	**0.53**
MP	**0.33**	**0.68**	**0.66**	**0.63**		**0.06**	**0.45**	**0.62**	−0.04	**0.64**	**0.49**	**0.61**	0.02	**0.73**	0.03	**0.76**	**0.55**	**0.72**	**0.23**
CS	**0.25**	**0.78**	**0.75**	**0.74**	0.06		**0.45**	**0.61**	0.00	**0.65**	**0.48**	**0.62**	0.03	**0.77**	−0.02	**0.84**	**0.58**	**0.80**	**0.19**
SL	**0.33**	**0.83**	**0.63**	**0.55**	**0.41**	**0.56**		**0.34**	**0.41**	**0.45**	**0.05**	**0.22**	**0.38**	**0.52**	**0.40**	**0.64**	0.00	**0.59**	**0.34**
AB	**0.24**	**0.52**	**0.55**	**0.51**	**0.17**	**0.18**	**0.32**		**0.60**	**0.51**	**0.36**	**0.41**	**0.57**	**0.57**	**0.58**	**0.72**	**0.34**	**0.69**	**0.51**
BR	**0.36**	**0.98**	**0.87**	**0.88**	**0.39**	**0.55**	**0.77**	0.12		**0.64**	**0.45**	**0.59**	−0.04	**0.79**	−0.05	**0.88**	**0.55**	**0.83**	0.15
BW	**0.54**	**0.93**	**0.92**	**0.92**	**0.78**	**0.87**	**0.89**	**0.64**	**0.99**		**0.46**	**0.52**	**0.55**	0.02	**0.56**	**0.83**	**0.51**	**0.79**	**0.51**
HB	**0.39**	**0.54**	**0.67**	**0.63**	**0.27**	**0.32**	**0.47**	**0.19**	**0.46**	**0.62**		**0.24**	**0.41**	**0.55**	**0.43**	**0.69**	−0.01	**0.63**	**0.35**
AL	**0.19**	**0.74**	**0.77**	**0.76**	**0.38**	**0.46**	**0.58**	**0.20**	**0.62**	**0.86**	**0.35**		**0.54**	**0.59**	**0.56**	**0.73**	**0.18**	**0.69**	**0.49**
FB	**0.57**	**0.99**	**0.94**	**0.94**	**0.80**	**0.89**	**0.92**	**0.61**	**0.99**	**0.99**	**0.76**	**0.49**		**0.63**	0.00	**0.68**	**0.46**	**0.62**	**0.17**
TC	**0.43**	**0.92**	**0.86**	**0.86**	**0.36**	**0.54**	**0.75**	**0.20**	**0.80**	**0.94**	**0.20**	**0.55**	**0.95**		**0.64**	**0.92**	**0.62**	**0.90**	**0.60**
DE	**0.25**	**0.66**	**0.70**	**0.67**	**0.18**	**0.26**	**0.46**	**0.04**	**0.35**	**0.78**	**0.15**	0.10	**0.71**	**0.11**		**0.71**	**0.49**	**0.64**	**0.16**
AF	**0.45**	**0.99**	**0.90**	**0.91**	**0.73**	**0.85**	**0.86**	**0.51**	**0.99**	**0.99**	**0.70**	**0.66**	**0.99**	**0.94**	**0.66**		**0.76**	0.02	**0.48**
DF	**0.23**	**0.98**	**0.88**	**0.88**	**0.24**	**0.44**	**0.77**	0.06	**0.95**	**0.99**	**0.22**	0.50	**0.99**	**0.31**	0.06	**1.00**		**0.71**	**0.40**
MN	**0.47**	**0.99**	**0.93**	**0.93**	**0.78**	**0.87**	**0.90**	**0.58**	**0.98**	**0.99**	**0.76**	**0.72**	**0.98**	**0.94**	**0.71**	**0.99**	**0.99**		**0.37**
KP	**0.29**	**0.99**	**0.89**	**0.89**	**0.64**	**0.77**	**0.81**	**0.43**	**0.98**	**0.99**	**0.61**	**0.42**	**0.97**	**0.91**	**0.51**	**1.00**	**1.00**	**0.98**	

Pairwise *Φst* values among *C. rossi* sampled localities for mitochondrial DNA (*COII*) below and nuclear intron (*EF1-α*) above the diagonal. Significant values (p < 0.05) are highlighted in bold. (Locality codes as in Table 1)

Fleas are considered generalist parasites and are thus predicted to show very little phylogeographic congruence with host genetic structure [38, 39, 44]. The present study provides the first evidence for the converse and, by making use of a comparative approach between two species, highlights the importance of the level of association between parasite and host in shaping genetic diversity across the landscape. The fur flea (*L. agrippinae*) which seems to have a narrower niche breath (i.e. narrower host range) [34, 35, 37], and spend longer time periods on the host, show three distinct mtDNA phylogeographic clades which are markedly congruent to the previously published regional vicariant biogeographic regions based on the patterns obtained in *Rhabdomys* [29]*, Micaelamys* [26], *Otomys* [28] and *Myosorex* [27] (see Additional file 1). Further support for the notion that the large scale dispersal of *L. agrippinae* is closely dependent on host movement can be obtained from the observation that the 95 % confidence interval of the time of divergence between the two major clades found in *L. agrippinae* (5.27 Ma; 95 % HPD interval: 2.31, 9.58 Ma) corresponds reasonably well with the timing of the divergence of the majority of rodent host lineages in the region [7]. In contrast, the more host opportunistic nest flea (*C. rossi*) show, in agreement with other phylogeographic studies on fleas [38, 39], virtually no congruence in phylogeographic patterns between host and parasite. The distinct clustering of *C. rossi* fleas sampled in the north east of the country (KP, MN, AF: Fig. 6a and Fig. 7) is not clearly depicted by longer branches in the Neighbor-Net analyses (Fig. 7c) and also not congruent with well documented biogeographic provinces. We argue that although this finding is interesting, it may simply be an artefact of the sampling distances between the various sampling sites.

The documented contact zone between *R. pumilio* and *R. dilectus* at Fort Beaufort ([29]; Fig. 1; Additional file 1) provides an interesting scenario to further explore host specificity among the two flea species (no contact zones have been described for the other hosts species sampled in this study). In the zone of contact, the two *Rhabdomys* species harbored distinct *L. agrippinae* mtDNA lineages represented by L1 (found exclusively on *R. pumilio*) and L2 (found exclusively on *R. dilectus*; Fig. 3b). In sharp contrast, the host opportunistic *C. rossi* show a high level of haplotype sharing among closely related localities in the same region (Figs. 6 and 7). *Rhabdomys* species is considered the principle hosts of *L. agrippinae* and *M. unisulcatus* is considered to be an auxiliary host, whereas *C. rossi* seems to have a larger host range [34, 35, 37]. Albeit based on a very small sample size, the tighter host association displayed by *L. agrippinae* might explain why we found distinct mtDNA lineages for *L. agrippinae* on *Rhabdomys* spp., but no differentiation in *C. rossi* in the contact zone.

When the intraspecific phylogeographic structures of *L. agrippinae* and *C. rossi* are compared to each other some more discrepancies are evident. First, our data provide additional support for the hypothesis that more generalist parasites will show higher levels of genetic diversity when compared to more specific parasites ([16]; Table 1). The connectivity among sampling sites is also markedly different between the two flea species. The more host specific fur flea *L. agrippinae,* show lower inter-populational divergence within clades (Fig. 3) when compared to the more host opportunistic nest flea, *C. rossi* (Fig. 6)*.* The same trend is evident when the nDNA data are considered (Figs. 4 and 7). The higher level of inter-populational divergences of *C. rossi* are best reflected by the large number of site changes among locality specific haplotypes (Fig. 6) and the numerous peaks on the landscape interpolation plots (Fig. 5b). These findings could be interpreted to be the direct result of differences in the dispersal abilities of the two fleas and can best be ascribed to differences in host association. Both parasites show isolation by distance but in both cases the correlation is extremely weak (indicated by the r values). In the case of the host specific fur flea, *L. agrippinae*, host movement provide hitchhiking possibilities within clades resulting in a higher level of connectivity among sampling sites. In the host opportunistic nest flea, *C. rossi*, the parasite has less opportunity to spread via host movement, resulting in a pattern of distantly related haplotypes at most sites. At specific geographic sites, it is possible that populations experience high levels of genetic drift that is homogenizing the locality specific signals [8, 41–43]. Long distance dispersals of the host opportunistic nest flea studied herein is a rare event and happen in a random way utilizing a wide niche breath (in the absence of strong isolation by distance), culminating in a pattern where some shared haplotypes are found on opposite ends of the geographic scale (for example see haplotype sharing between the group of localities with different shades of blue (FB, AL, TC, HB, DE) and geographically distant localities (BR and also DF; Fig. 6).

From our study it was also evident that *L. agrippinae* occurred at a lower prevalence than *C. rossi* overall and the pattern was also apparent at most localities where the distribution of the two species overlap. The same trend in prevalence and abundance for *L. agrippinae* and *C. rossi* was recorded in previous studies in South Africa [35, 37, 45]. The two flea species differ in terms of body length with *L. agrippinae* being larger (on average 3650 μm) compared to *C. rossi* (on average 1750 μm) ([46]; also supported by body size index in [35]). Studies on free-living taxa and more recently on ectoparasitic mites of small mammals recorded a negative relationship between body size and abundance [47–49]. In the latter study it was suggested that the pattern may be due to higher host-induced mortality (grooming) associated with larger bodied ectoparasites [49]. *Listropsylla agrippinae* is almost twice the size of *C. rossi* and it is possible that host grooming (allo- and autogrooming) resulted in lower *L. agrippinae* prevalence. Interestingly, in the present study the difference in body size between the two flea species seem to support their level of host specificity. Studies on microbes and diatoms and more recently on ectoparasitic mites recorded a negative relationship between body size and niche breadth [49–51]. It appears that smaller bodied species are more adaptable to environmental fluctuations which results in a larger niche breath or in the case of parasites a larger host range. This is in contrast to larger species that have a smaller niche breath/host range due to narrower tolerance levels [49–51].

If prevalence is positively correlated to abundance in fleas [52, 53], then it could be argued that the higher abundance (and niche breath) of the more host opportunistic nest flea, *C. rossi,* on the hosts will facilitate dispersal among sampling sites. This pattern is however contrary to what we found in the present study where *C. rossi* is more structured between sampling sites when compared to *L. agrippinae*. One possible explanation for this may relate to differences in the effective population sizes between the two flea species. A higher genetic diversity, as found in *C. rossi,* is expected for populations with larger effective population sizes [41, 44] and it is furthermore reasonable to predict that nest fleas will predominantly have higher abundances in the nests of their hosts [32, 39]. Given the nest bound nature of *C. rossi,* the number of dispersing individuals on the hosts may thus not be large enough to overcome the effects of drift when introduced into the nests of the hosts elsewhere (containing a large population of local genotypes in the new environment). In contrast, for *L. agrippinae,* the comparatively lower effective population sizes can facilitate the signature of more haplotype sharing among localities within clades.

## Conclusions

In the light of re-emerging flea borne diseases worldwide, it is important to have a thorough understanding of the mechanisms that are involved in shaping flea distribution and movement [22]. From our study it is evident that host association (microhabitat preference and host specificity) plays an important role in flea dispersal and subsequent gene flow within and between geographic locations. This study also provides the first evidence of congruent phylogeographic vicariant patterns between a generalist parasitic flea and its hosts. Unfortunately our conclusions are based on a single study comprising two distinct life histories. More parasite taxa and gene fragments needs to be evaluated in order to formulate stronger hypotheses in this regard.

## Methods

### Sample collection

Small mammal trapping was performed during 2010–2013 at 20 localities (natural areas and low density grazing farms) in South Africa (Fig. 1a; Table 5). Baited Sherman-type live traps were set in a line transect and sampling varied between 4 to 7 days per locality. All adult specimens of species that are listed as potential hosts for the two flea species were selected and juveniles were released at the trap site. Trapped animals were placed in a plastic bag before they were euthanized with sodium pentobarbital (200 mg/kg; ethical approval reference number SU-ACUM11-00004). The bodies were brushed over a white plastic tray and all fleas were collected. The brush was inspected and cleaned (using 96 % ethanol) after each animal was processed and new brushes were used for each host species at each locality. Individual fleas were placed in separate tubes filled with 96 % ethanol. Before DNA extraction, *L. agrippinae* and *C. rossi* individuals were preliminary identified using a Leica stereoscopic microscope (Leica Microsystems, Wetzlar, Germany) and the taxonomic key of [34]. After DNA extraction the exoskeletons of all extracted fleas were mounted (see [35]) and a thorough morphological identification was done under a Leica DM 3000 light microscope (Leica Microsystems, Wetzlar, Germany) using the key of [34]. Most of the host species that were trapped during the study are quite common and widely distributed throughout South Africa. As a result voucher specimens of the species are readily available in several museums. In the case of the fleas, voucher specimens will be deposited in the Museum of the Department of Conservation Ecology and Entomology (Stellenbosch University) and the National Flea Collection in Johannesburg (both in South Africa).

**Table 5:** Locality information

Province	Locality	Code	Geographic coordinates	*L. agrippinae* (n)		*C. rossi* (n)	
				COII	EF1-*α*	COII	EF1-α
*Western Cape*	Anysberg	AB	−33.46 S 20.59 E	5	1	14	13
	Beaufort West	BW	−32.22 S 22.80 E	13	7	14	6
	Buffeljagsrivier	BR	−34.05 S 20.53 E	6	0	10	9
	Kanu	CS	−33.95 S 18.83 E	10	5	7	6
	Mooiplaas	MP	−33.92 S 18.75 E	4	4	11	8
	Vanrhynsdorp	VR	−31.73 S 18.77 E	5	3	15	11
*Northern Cape*	Dronfield	DF	−28.74 S 24.77 E	*	*	4	3
	Garies	GS	−30.43 S 17.89 E	10	4	5	4
	Loeriesfontein	LF	−30.95 S 19.44 E	15	6	16	14
	Springbok	GP	−29.70 S 18.03 E	14	6	3	2
	Sutherland	SL	−32.40 S 20.90 E	14	4	8	8
	Three Sisters	TS	−31.89 S 23.15 E	10	2	*	*
*Eastern Cape*	Alice	AL	−32.79 S 26.85 E	*	*	5	2
	Dohne	DE	−32.53 S 27.46 E	11	2	10	5
	Fort Beaufort	FB	−32.78 S 26.63 E	9	3	17	8
	Hogsback	HB	−32.59 S 26.92 E	*	*	17	10
	The Croft	TC	−32.55 S 27.37 E	*	*	11	6
*Gauteng*	Kaalplaas	KP	−25.63 S 28.17 E	*	*	5	5
*North West*	Mooinooi	MN	−25.47 S 27.33 E	*	*	8	2
*KwaZulu-Natal*	Albert Falls	AF	−29.47 S 30.40 E	*	*	9	5
6 provinces	20 localities			126	47	189	127

Locality information from where specimens were obtained for each of the two flea species, with codes corresponding to Fig. 1

### DNA extraction, amplification and sequencing

Total genomic DNA was extracted with a Qaigen, DNeasy® Blood and Tissue kit (Qaigen, Valencia, CA, USA) following the protocol of the manufacturer. Whole flea specimens were placed in the extraction buffer containing Proteinase K (600 mAU/ml solution or 40 mAU/mg protein), and digested at 56 °C overnight. After digestion, flea exoskeletons were removed for identification purposes (see above).

Polymerase Chain Reactions (PCR) and sequencing were performed on the mitochondrial Cytochrome Oxidase II (*COII*) gene and the nuclear intron Elongation Factor 1 alpha (*EF1-*α) using published primers ([54, 55]; Table 5; Additional file 3) Nuclear and mitochondrial regions were amplified using a GeneAmp® PCR 2700 thermal cycler (Applied Biosystems, Foster City, CA, USA). Mitochondrial *COII* and nuclear *EF1-*α regions were amplified following standard procedures (Additional file 3). Sequencing was performed on an ABI 3730 XL DNA analyzer (Applied Biosystems) using BigDye termination chemistry (version 3.1, Applied Biosystems).

### Alignment and phylogenetic analyses

Sequences for each gene fragment were edited and aligned using BioEdit Sequence Alignment editor 7.2.5 [56]. Mitochondrial sequences were translated into amino acids using EMBOSStranseq (www.ebi.ac.uk/Tools/st/emboss_transeq) to confirm functionality. Heterozygous positions in the nuclear fragments were resolved in DNASP 5 [57] using PHASE 2.1.1 [58, 59]. The algorithm was run for 1000 generations with a thinning interval of 1 and burn-in of 100 generations. Phases with a 0.9 probability or higher were considered resolved and the analysis was performed three times to see if there was any significant difference in results between runs [58].

### Diversity indices and population level analyses

Nucleotide diversity (π) and haplotype diversity (*h*) values were obtained using DNASP 5 [57]. The evolutionary relationships between haplotypes were investigated by constructing statistical parsimony networks with 95 % confidence intervals in TCS 1.21 [60]. The best-fit models of sequence evolution were determined for each fragment under the AICc [61, 62] in jModelTest 2.1.4 [63, 64]. To further gain an evolutionary perspective on the association between clusters, individual HKY-corrected networks of each flea species were drawn for mitochondrial *COII* and nuclear *EF1-*α using the Neighbor-Net method [65] implemented in SplitsTree 4.5 [66]. HKY-corrected sequence distances among mitochondrial *COII* and nuclear *EF1-*α were calculated using PAUP* v4.0b10 [67]. To investigate population structure without a-priori assumptions, a Bayesian Analyses of Population Structure (BAPS) was performed in BAPS 6.0 [68] on the mtDNA data. Spatial genetic mixture analyses of individuals and of groups were performed independently using a vector of maximum K values (each replicated 5 times; [68, 69]). Analysis of molecular variance (AMOVA; [70] and pairwise Φ*st* statistics between sampled populations were performed in ARLEQUIN 3.5.1.2 [71]. Only sampling localities with more than 5 individuals were included. The AMOVA higher level group differentiations were defined based on the subclusters obtained in the BAPS analysis (only sampling localities with more than 5 individuals were used). Mantel tests [72] were performed to test for isolation by distance in ALLELES IN SPACE [73]. Spatial genetic structure was further explored by making use of genetic landscape shape interpolation surface plots constructed in ALLELES IN SPACE [73].

### Dating main phylogenetic events

We used a relaxed exponential Bayesian molecular clock as implemented in BEAST 2.1.3 [74] to estimate divergence time between clusters. Siphonaptera lacks a useable fossil record and as a calibration point we employed the 2.3 % per million years estimated for various arthropod taxa [75, 76]. The HKY + G model was used and the birth-death process of speciation with exponential priors specified. MCMC simulation ran for 20 million generations, sampling every 10 000 generations for each of the two runs performed. Convergence and mixing were assessed in Tracer 1.6 [74] and the first 25 % of the trees were discarded as burn-in. A maximum clade credibility tree was produced in TreeAnnotator 2.1.3 [74].

## Availability of supporting data

DNA sequence data are available in GenBank [GenBank: KR 263182–263844].

## Additional files

Additional file 1:
**Small mammal biogeographic patterns.** Maps indicating published biogeographic patterns directly obtained for **a**) *Rhabdomys* [29], **b**) *Micaelamys* [26], **c**) *Otomys* [28] and **d**) *Myosorex* [27]. Additional file 2:
**Host identity, abundance and parasite prevalence.** Host identity and abundance, parasite prevalence and the number of specimens sequenced for each flea species per locality. *Could not determine which host the samples were sequenced from. Additional file 3:
**Primer and PCR specifications.docx.** Primers used for PCR amplification of mitochondrial and nuclear genes for the two flea species. *COII* amplification consisted of a denaturation cycle of 1 min at 95 °C followed by a 10 cycle loop of 1 min at 95 °C, 45 °C, and 72 °C, respectively. A 30 cycle loop was then performed with denaturation for 1 min at 93 °C followed by annealing for 1 min at the primer specific temperature, and 1 min extension at 72 °C, followed by a final extension period of 5 min at 72 °C. General PCR cycling conditions for the *EF1-*α region included an initial denaturation of 5 min at 94 °C followed by 40 cycles of 30 s denaturation at 94 °C, 45 s annealing at primer specific temperature, and 1 min extension at 72 °C, followed by a final extension period of 7 min at 72 °C. *Primers from [54].
