# Supplementary figures and images for: Comparative phylogeography between two generalist flea species reveal a complex interaction between parasite life history and host vicariance: parasite-host association matters
Source: BMC Evol Biol. 2015 Jun 10;15:105. doi: 10.1186/s12862-015-0389-y (PMC4460865; doi:10.1186/s12862-015-0389-y)

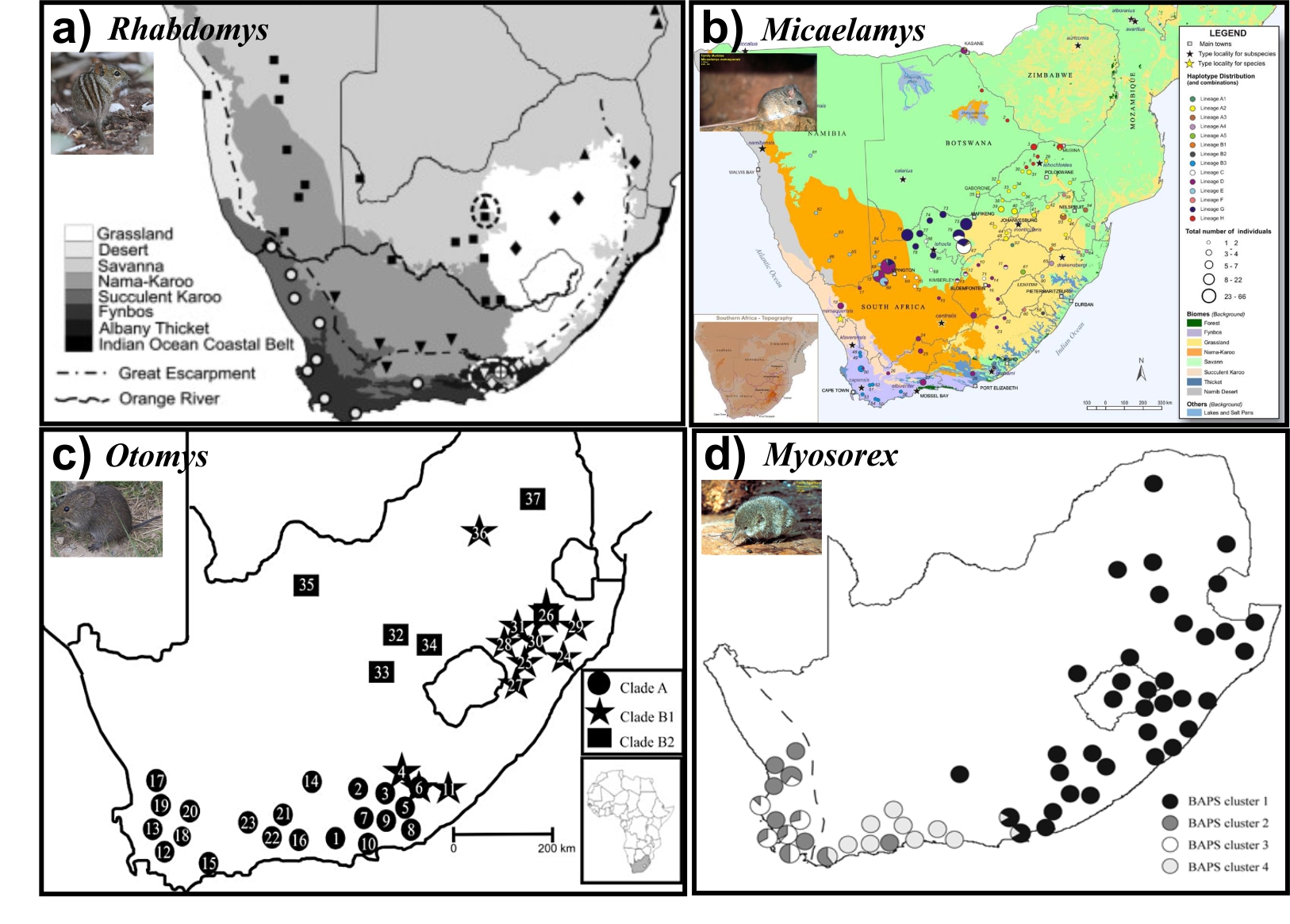

Supplement: Additional file 1: — Small mammal biogeographic patterns. Maps indicating published biogeographic patterns directly obtained for a) Rhabdomys [29], b) Micaelamys [26], c) Otomys [28] and d) Myosorex [27]. [file 12862_2015_389_MOESM1_ESM.jpeg]
