# Supplementary material for: Comparative phylogeography between two generalist flea species reveal a complex interaction between parasite life history and host vicariance: parasite-host association matters
Source: BMC Evol Biol. 2015 Jun 10;15:105. doi: 10.1186/s12862-015-0389-y (PMC4460865; doi:10.1186/s12862-015-0389-y)
Supplement: Additional file 2: — Host identity, abundance and parasite prevalence. Host identity and abundance, parasite prevalence and the number of specimens sequenced for each flea species per locality. *Could not determine which host the samples were sequenced from. [file 12862_2015_389_MOESM2_ESM.docx]

**Additional file 2** Host identity and abundance, parasite prevalence and the number of specimens sequenced for each flea species per locality. *Could not determine which host the samples were sequenced from.

| **Province** | **Locality** | **Code** | **Geographic coordinates** | **Host** | **Host (*n*)** | ***Listropsylla agrippinae*** | | ***Chiastopsylla rossi*** | |
| --- | --- | --- | --- | --- | --- | --- | --- | --- | --- |
|  |  |  |  |  |  | **Prevalence (%)** | **Sequenced (n)** | **Prevalence (%)** | **Sequenced (n)** |
| *Western Cape* | Anysberg | AB | -33.46 S 20.59 E | *Rhabdomys pumilio* | 33 | 3 | 4 | 42 | 12 |
|  |  |  |  | *Micealamys namaquensis* | 6 | 17 | 1 | 0 | 0 |
|  |  |  |  | *Otomys irroratus* | 1 | 100 | 0 | 100 | 2 |
|  |  |  |  | *Myotomys unisulcatus* | 10 | 0 | 0 | 0 | 0 |
|  |  |  |  | *Elephantulus edwardii* | 1 | 0 | 0 | 0 | 0 |
|  |  |  |  | Total | 51 | 6 | 5 | 29 | 14 |
|  |  |  |  |  |  |  |  |  |  |
|  | Beaufort West | BW | -32.22 S 22.80 E | *Rhabdomys intermedius* | 33 | 6 | 10 | 36 | 10 |
|  |  |  |  | *Micealamys namaquensis* | 6 | 33 | 2 | 0 | 0 |
|  |  |  |  | *Otomys irroratus* | 4 | 100 | 1 | 75 | 4 |
|  |  |  |  | *Myotomys unisulcatus* | 4 | 0 | 0 | 0 | 0 |
|  |  |  |  | Shrew spp. | 2 | 0 | 0 | 0 | 0 |
|  |  |  |  | Total | 49 | 12 | 13 | 31 | 14 |
|  |  |  |  |  |  |  |  |  |  |
|  | Buffeljagsrivier | BR | -34.05 S 20.53 E | *Rhabdomys pumilio* | 27 | 19 | 6 | 59 | 8 |
|  |  |  |  | *Otomys irroratus* | 8 | 0 | 0 | 25 | 2 |
|  |  |  |  | Shrew spp. | 1 | 0 | 0 | 0 | 0 |
|  |  |  |  | Total | 36 | 14 | 6 | 50 | 10 |
|  |  |  |  |  |  |  |  |  |  |
|  | Kanu | CS | -33.95 S 18.83 E | *Rhabdomys pumilio* | 73 | 12 | 10 | 36 | 7 |
|  |  |  |  | *Micealamys namaquensis* | 5 | 0 | 0 | 0 | 0 |
|  |  |  |  | *Otomys irroratus* | 10 | 0 | 0 | 0 | 0 |
|  |  |  |  | Shrew spp. | 6 | 0 | 0 | 0 | 0 |
|  |  |  |  | *Mus minutoides* | 9 | 0 | 0 | 0 | 0 |
|  |  |  |  | Total | 103 | 9 | 10 | 25 | 7 |
|  |  |  |  |  |  |  |  |  |  |
|  | Mooiplaas | MP | -33.92 S 18.75 E | *Rhabdomys pumilio* | 35 | 11 | 4 | 34 | 9 |
|  |  |  |  | *Otomys irroratus* | 1 | 0 | 0 | 0 | 0 |
|  |  |  |  | *Elephantulus edwardii* | 1 | 0 | 0 | 100 | 2 |
|  |  |  |  | *Myotomys unisulcatus* | 5 | 0 | 0 | 0 | 0 |
|  |  |  |  | Total | 42 | 10 | 4 | 31 | 11 |
|  |  |  |  |  |  |  |  |  |  |
|  | Vanrhynsdorp | VR | -31.73 S 18.77 E | *Rhabdomys pumilio* | 25 | 8 | 5 | 56 | 15 |
|  |  |  |  | *Micealamys namaquensis* | 4 | 0 | 0 | 0 | 0 |
|  |  |  |  | *Otomys irroratus* | 1 | 0 | 0 | 100 | 0 |
|  |  |  |  | *Mus musculus* | 1 | 0 | 0 | 0 | 0 |
|  |  |  |  | Total | 31 | 6 | 5 | 48 | 15 |
|  |  |  |  |  |  |  |  |  |  |
| *Northern Cape* | Dronfield | DF | -28.74 S 24.77 E | *Rhabdomys intermedius* | 32 | 9 | 0 | 34 | 4 |
|  |  |  |  | *Tatera leucogaster* | 1 | 0 | 0 | 0 | 0 |
|  |  |  |  | *Rattus rattus* | 1 | 0 | 0 | 0 | 0 |
|  |  |  |  | *Mastomys coucha* | 2 | 0 | 0 | 0 | 0 |
|  |  |  |  | Total | 36 | 8 | 0 | 31 | 4 |
|  |  |  |  |  |  |  |  |  |  |
|  | Garies | GS | -30.43 S 17.89 E | *Rhabdomys pumilio* | 27 | 19 | 6 | 26 | 5 |
|  |  |  |  | *Micealamys namaquensis* | 15 | 13 | 4 | 7 | 0 |
|  |  |  |  | *Myotomys unisulcatus* | 2 | 0 | 0 | 0 | 0 |
|  |  |  |  | *Elephantulus edwardii* | 2 | 0 | 0 | 0 | 0 |
|  |  |  |  | *Gerbillurus paeba* | 1 | 0 | 0 | 0 | 0 |
|  |  |  |  | Total | 47 | 15 | 10 | 17 | 5 |
|  |  |  |  |  |  |  |  |  |  |
|  | Loeriesfontein | LF | -30.95 S 19.44 E | *Rhabdomys intermedius* | 31 | 48 | 12 | 48 | 15 |
|  |  |  |  | *Micealamys namaquensis* | 16 | 31 | 1 | 0 | 0 |
|  |  |  |  | *Myotomys unisulcatus* | 5 | 40 | 2 | 40 | 1 |
|  |  |  |  | *Elephantulus edwardii* | 3 | 0 | 0 | 0 | 0 |
|  |  |  |  | Total | 55 | 40 | 15 | 31 | 16 |
|  |  |  |  |  |  |  |  |  |  |
|  | Springbok | GP | -29.70 S 18.03 E | *Rhabdomys pumilio* | 56 | 23 | 12 | 2 | 1 |
|  |  |  |  | *Micealamys namaquensis* | 31 | 0 | 0 | 6 | 0 |
|  |  |  |  | *Myotomys unisulcatus* | 17 | 6 | 2 | 18 | 2 |
|  |  |  |  | *Mus musculus* | 1 | 0 | 0 | 0 | 0 |
|  |  |  |  | *Elephantulus edwardii* | 5 | 0 | 0 | 0 | 0 |
|  |  |  |  | *Desmodillus auricularis* | 4 | 0 | 0 | 0 | 0 |
|  |  |  |  | *Parotomys brantsii* | 2 | 0 | 0 | 0 | 0 |
|  |  |  |  | Total | 116 | 12 | 14 | 5 | 3 |
|  |  |  |  |  |  |  |  |  |  |
|  | Sutherland | SL | -32.40 S 20.90 E | *Rhabdomys intermedius* | 25 | 32 | 14 | 40 | 8 |
|  |  |  |  | *Myotomys unisulcatus* | 9 | 33 | 0 | 67 | 0 |
|  |  |  |  | *Gerbillurus paeba* | 1 | 0 | 0 | 0 | 0 |
|  |  |  |  | Total | 35 | 31 | 14 | 46 | 8 |
|  |  |  |  |  |  |  |  |  |  |
|  | Three Sisters | TS | -31.89 S 23.15 E | *Rhabdomys intermedius* | 5 | 20 | 1 | 20 | 0 |
|  |  |  |  | *Micealamys namaquensis* | 6 | 17 | 2 | 0 | 0 |
|  |  |  |  | *Myotomys unisulcatus* | 9 | 22 | 7 | 0 | 0 |
|  |  |  |  | *Mus musculus* | 4 | 0 | 0 | 0 | 0 |
|  |  |  |  | *Gerbillurus paeba* | 1 | 0 | 0 | 0 | 0 |
|  |  |  |  | Shrew spp. | 2 | 0 | 0 | 0 | 0 |
|  |  |  |  | Total | 27 | 15 | 10 | 4 | 0 |
|  |  |  |  |  |  |  |  |  |  |
| *Eastern Cape* | Alice | AL | -32.79 S 26.85 E | *Rhabdomys dilectus* | 6 | 0 | 0 | 17 | 3 |
|  |  |  |  | *Otomys irroratus* | 8 | 0 | 0 | 13 | 1 |
|  |  |  |  | *Rattus rattus* | 2 | 0 | 0 | 0 | 0 |
|  |  |  |  | *Mastomys natalensis* | 15 | 0 | 0 | 7 | 1 |
|  |  |  |  | Total | 31 | 0 | 0 | 10 | 5 |
|  |  |  |  |  |  |  |  |  |  |
|  | Dohne | DE | -32.53 S 27.46 E | *Rhabdomys dilectus* | 30 | 30 | 10 | 30 | 5 |
|  |  |  |  | *Otomys irroratus* | 8 | 13 | 1 | 75 | 2 |
|  |  |  |  | Shrew spp. | 6 | 0 | 0 | 17 | 3 |
|  |  |  |  | Total | 44 | 23 | 11 | 36 | 10 |
|  |  |  |  |  |  |  |  |  |  |
|  | Fort Beaufort | FB | -32.78 S 26.63 E | *Rhabdomys pumilio* | 8 | 25 | 3 | 38 | 4 |
|  |  |  |  | *Rhabdomys dilectus* | 8 | 50 | 5 | 88 | 6 |
|  |  |  |  | *Micealamys namaquensis* | 16 | 13 | 1 | 13 | 1 |
|  |  |  |  | *Otomys irroratus* | 6 | 0 | 0 | 33 | 4 |
|  |  |  |  | *Mus musculus* | 2 | 0 | 0 | 0 | 0 |
|  |  |  |  | Shrew spp. | 2 | 0 | 0 | 50 | 1 |
|  |  |  |  | Total | 42 | 19 | 9 | 36 | 16 |
|  |  |  |  |  |  |  |  |  |  |
|  | Hogsback | HB | -32.59 S 26.92 E | *Rhabdomys dilectus* | 10 | 30 | 0 | 30 | 3 |
|  |  |  |  | *Otomys irroratus* | 2 | 50 | 0 | 100 | 13 |
|  |  |  |  | *Mus musculus* | 4 | 0 | 0 | 25 | 1 |
|  |  |  |  | *Rattus rattus* | 1 | 0 | 0 | 0 | 0 |
|  |  |  |  | Shrew spp. | 1 | 0 | 0 | 0 | 0 |
|  |  |  |  | Total | 18 | 22 | 0 | 33 | 17 |
|  |  |  |  |  |  |  |  |  |  |
|  | The Croft | TC | -32.55 S 27.37 E | *Rhabdomys dilectus* | 25 | 0 | 0 | 16 | 4 |
|  |  |  |  | *Otomys irroratus* | 15 | 0 | 0 | 33 | 6 |
|  |  |  |  | Shrew spp. | 3 | 0 | 0 | 33 | 1 |
|  |  |  |  | Total | 43 | 0 | 0 | 23 | 11 |
|  |  |  |  |  |  |  |  |  |  |
| *Gauteng* | Kaalplaas* | KP | -25.63 S 28.17 E | *Rhabdomys dilectus* | 97 | * | * | * | * |
|  |  |  |  | *Otomys irroratus* | 1 | * | * | * | * |
|  |  |  |  | *Mus musculus* | 1 | * | * | * | * |
|  |  |  |  | *Mastomys natalensis* | 44 | * | * | * | * |
|  |  |  |  | Shrew spp. | 11 | * | * | * | * |
|  |  |  |  | *Dasymys* spp. | 4 | * | * | * | * |
|  |  |  |  | Total | 158 | 0 | 0 | 9 | 5 |
|  |  |  |  |  |  |  |  |  |  |
| *North West* | Mooinooi* | MN | -25.47 S 27.33 E | *Micealamys chrysophilus* | 8 | * | * | * | * |
|  |  |  |  | *Mus musculus* | 7 | * | * | * | * |
|  |  |  |  | *Mastomys natalensis* | 21 | * | * | * | * |
|  |  |  |  | Shrew spp. | 3 | * | * | * | * |
|  |  |  |  | *Lemniscomous rosalia* | 14 | * | * | * | * |
|  |  |  |  | *Steatomys pratensis* | 10 | * | * | * | * |
|  |  |  |  | Total | 63 | 0 | 0 | 13 | 8 |
|  |  |  |  |  |  |  |  |  |  |
| *KwaZulu-Natal* | Albert Falls | AF | -29.47 S 30.40 E | *Mastomys natalensis* | 26 | 0 | 0 | 15 | 5 |
|  |  |  |  | *Otomys irroratus* | 2 | 0 | 0 | 100 | 4 |
|  |  |  |  | Shrew spp. | 1 | 0 | 0 | 0 | 0 |
|  |  |  |  | Total | 29 | 0 | 0 | 21 | 9 |
