# Supplementary material for: Comparative phylogeography between two generalist flea species reveal a complex interaction between parasite life history and host vicariance: parasite-host association matters
Source: BMC Evol Biol. 2015 Jun 10;15:105. doi: 10.1186/s12862-015-0389-y (PMC4460865; doi:10.1186/s12862-015-0389-y)
Supplement: Additional file 3: — Primer and PCR specifications.docx. Primers used for PCR amplification of mitochondrial and nuclear genes for the two flea species. COII amplification consisted of a denaturation cycle of 1 min at 95 °C followed by a 10 cycle loop of 1 min at 95 °C, 45 °C, and 72 °C, respectively. A 30 cycle loop was then performed with denaturation for 1 min at 93 °C followed by annealing for 1 min at the primer specific temperature, and 1 min extension at 72 °C, followed by a final extension period of 5 min at 72 °C. General PCR cycling conditions for the EF1-α region included an initial denaturation of 5 min at 94 °C followed by 40 cycles of 30 s denaturation at 94 °C, 45 s annealing at primer specific temperature, and 1 min extension at 72 °C, followed by a final extension period of 7 min at 72 °C. *Primers from [54]. [file 12862_2015_389_MOESM3_ESM.docx]

**Additional file 3** Primers used for PCR amplification of mitochondrial and nuclear genes for the two flea species. *COII* amplification consisted of a denaturation cycle of 1 min at 95°C followed by a 10 cycle loop of 1 min at 95°C, 45°C, and 72°C, respectively. A 30 cycle loop was then performed with denaturation for 1min at 93°C followed by annealing for 1 min at the primer specific temperature, and 1 min extension at 72°C, followed by a final extension period of 5 min at 72°C. General PCR cycling conditions for the *EF1-α* region included an initial denaturation of 5 min at 94°C followed by 40 cycles of 30 s denaturation at 94°C, 45 s annealing at primer specific temperature, and 1 min extension at 72°C, followed by a final extension period of 7 min at 72°C. *Primers from [51].

| **Taxon** | **Marker** | **Primer** | **F/R** | **Sequence (5'-3')** | **Annealing temperature (°C)** |
| --- | --- | --- | --- | --- | --- |
| *L agrippinae* | COII | COII-2a* | F | ATAGAKCWTCYCCHTTAATAGAACA | 50-52°C |
|  |  | COII-9b* | R | GTACTTGCTTTCAGTCATCTWATG | 50-52°C |
|  | EF1-α | ForlagNEW | F | TTGGATGGCACCAAGTTGAC | 59.6°C |
|  |  | RevlagNEW | R | TGGCTTTCACTTTGGGAGTC | 59.6°C |
| *C rossi* | COII | COII-2a* | F | ATAGAKCWTCYCCHTTAATAGAACA | 50-52°C |
|  |  | COII-9b* | R | GTACTTGCTTTCAGTCATCTWATG | 50-52°C |
|  | EF1-α | EF-1a M 44-1* | F | GCTGAGCGYGARCGTGGTATCAC | 59.6°C |
|  |  | EF-1a rcM 4.0* | R | ACAGVCACKGTYTGYCTCATRTC | 59.6°C |
